# Design and Construction of UAV-Based Measurement System for Water Hyperspectral Remote-Sensing Reflectance

**DOI:** 10.3390/s25092879

**Published:** 2025-05-02

**Authors:** Haohui Zeng, Xianqiang He, Yan Bai, Fang Gong, Difeng Wang, Xuan Zhang

**Affiliations:** 1 Ocean College, Zhejiang University, Zhoushan 316021, China; 2 State Key Laboratory of Satellite Ocean Environment Dynamics, Second Institute of Oceanography, Ministry of Natural Resources, Hangzhou 310012, China

**Keywords:** UAV, water hyperspectral observation, radiometric calibration, water color remote sensing, remote-sensing reflectance

## Abstract

Acquiring a large number of in situ water spectral measurements is fundamental for constructing water color remote-sensing retrieval models and validating the accuracy of water color remote-sensing products. However, traditional manual site-based water spectral measurements are time-consuming and labor-intensive, resulting in an insufficient number of in situ water spectral samples to date. To resolve this issue, this study develops an unmanned aerial vehicle-based hyperspectral remote-sensing reflectance measurement system (UAV-RRS) capable of continuous on-the-move water spectral measurements. This paper provides a detailed introduction to the system components and conducts precise experiments on the correction and calibration of the spectral sensors. Using this system, an in situ–UAV–satellite multi-source remote-sensing reflectance comparison experiment was conducted in the middle reaches of the Qiantang River, East China, to evaluate the accuracy and reliability of UAV-RRS and extend the analysis to satellite data across different spatial scales. The results demonstrate that, in small-scale water bodies, UAV-RRS achieves higher spatial precision and spectral accuracy, offering a valuable solution for high-precision, low-altitude continuous water body observations.

## 1. Introduction

Remote-sensing reflectance (*R_rs_*, units: $sr^{-1}$) is defined as the ratio of the water-leaving radiance (*L_w_*, units: $\mu Wcm^{-2}sr^{-1}nm^{-1}$) to the downwelling irradiance just above the water surface (*E_s_*, units: $\mu Wcm^{-2}nm^{-1}$) [1]. *R_rs_* quantifies the spectral “brightness” of water as seen by a remote sensor, normalized to incoming solar irradiance, which removes variations caused by changing illumination conditions. Due to the direct correlation between *R_rs_* and inherent optical properties (IOPs), such as absorption (*a*) and backscattering (*b_b_*) coefficients, *R_rs_* serves as a crucial parameter in the development of water color remote-sensing retrieval models, including chlorophyll-a (Chl-a), total suspended matter (TSM), colored dissolved organic matter (CDOM) concentration [2], and submerged macrophyte detection or bottom-depth measurement for shallow waters [3,4].

The in situ measurement of water’s apparent optical properties (AOPs) including *R_rs_* can be categorized into two primary approaches: profiling-based measurements [5] and above-water measurements [6]. Profiling-based methods employ submerged instruments to capture the vertical distribution of the aquatic light field, which is then extrapolated to derive optical signals at the water surface. However, these methods are often complex, requiring the cumbersome deployment of large-scale instruments, and are only effective in deep-water regions. In contrast, above-water measurements, like terrestrial spectral observations, utilize rigorously calibrated instruments to acquire water reflectance. Given that water surfaces are inherently non-Lambertian and exhibit directionally sensitive reflectance [7], precise measurement geometries and integration times must be carefully configured to obtain an accurate *R_rs_*. For optically complex Case 2 waters, above-water methods currently serve as the only effective approach for measuring *R_rs_*.

In the field of optical remote sensing, large-scale applications are currently dominated by satellite remote sensing. Water color satellites enable extensive and high-frequency observations of global water bodies, providing long-term monitoring capabilities for large-scale water bodies such as lakes [8], major rivers [9], coastal zones, and nearshore regions [10,11]. However, for specific high-precision studies of inland Case 2 water bodies, such as narrow rivers or small lakes, satellite observations often suffer from insufficient spatial resolution or lower data update frequency. Additionally, due to the complex atmospheric aerosol characteristics in nearshore regions, atmospheric correction for Case 2 water bodies is more challenging than for Case 1 waters and is more significantly affected by terrestrial influences [12].

Limitations in the spectral, spatial, and temporal resolutions of satellite sensors restrict their ability to effectively monitor fine-scale hydrological and aquatic dynamics [13]. In situ spectral measurements remain the primary means of acquiring high-precision water optical properties. Several specialized spectrometers have been developed for above-water *R_rs_* measurements, such as the Analytical Spectral Devices (ASDs) from Malvern Panalytical [14], the RAMSES series from TriOS [15], and HyperOCR from Sea-Bird Scientific [16]. Additionally, ground-based observation systems have been explored in recent studies. Sun et al. [17] developed a proximal hyperspectral imager, installed at 4–5 m above the water surface, enabling continuous spectral observation of river cross-sections. Leeuw et al. [18] introduced a mobile application, HydroColor, which utilizes a smartphone camera as a three-band radiometer and, when combined with an 18% reflectance gray card, can estimate *R_rs_* with an accuracy within 26% of a precision radiometer. Coqué et al. [19] designed a portable buoy system based on the Skylight-Blocked Approach (SBA) to optimize water-leaving radiance measurements by maintaining ideal observation geometry. These existing field spectrometers and measurement systems are well suited for ground- or ship-based automatic or manual data collection. However, their bulkiness and complex deployment often limit their spatial coverage, resulting in sparse data collection that cannot effectively capture the spatial variability of aquatic environments.

With advancements in unmanned aerial vehicle (UAV) technology and miniaturized spectrometers, UAV-based remote sensing has emerged as a promising approach for acquiring high-accuracy water spectral data. Current UAV spectral sensors mainly consist of multispectral and hyperspectral imaging systems, including the Specim AFX series from Konica Minolta [20], RedEdge-MX from MicaSense [21], the Mjolnir hyperspectral imaging system from HySpex [22], and the GaiaSky-mini hyperspectral imaging camera from Zolix [23]. As a near-surface observation platform, UAV remote sensing effectively addresses the low-data-density issue associated with ground-based point measurements, while also offering greater flexibility than satellite imagery by overcoming temporal constraints. The lower flight altitude of UAVs provides highly precise spectral data, contributing to the development of water color remote-sensing models and the validation of water color products. UAV remote sensing has been widely applied in various water quality and aquatic ecosystem monitoring applications, including water quality parameter estimation [24,25,26], water transparency assessment [27,28], shallow water bathymetry [29,30], algal bloom monitoring [31], pollutant tracking [32], and hydrodynamic process analysis [33,34,35].

For quantitative water color remote sensing, ensuring the radiometric accuracy of spectral sensors is vital, as the reliability of the derived water color products largely depends on spectral measurement precision. However, UAV-based water remote-sensing studies often overlook the accuracy of acquired signals or images. Hyperspectral imagers typically use prisms, gratings, or filter arrays for spectral dispersion. These dispersing components may cause a reduction in spectral resolution when light is dispersed across a wide field of view (e.g., uneven dispersion or optical path differences) [36]. Furthermore, hyperspectral imagers need to balance both two-dimensional spatial imaging and one-dimensional spectral acquisition, leading to higher optical system complexity, potential light flux loss, and stray light interference. In many cases, UAV-mounted multispectral and hyperspectral cameras capture only digital number (DN) values rather than absolute radiometric quantities with physical optical significance. Some studies have attempted to correct this by deploying diffuse reflectance plates of known reflectance on the ground to measure reference radiance signals [37], but this introduces issues related to geometry mismatch between target observation and reflectance calibration, as environmental conditions are likely to have changed between the reference and the target measurement. Moreover, hyperspectral imaging systems typically have non-ideal photon acceptance angles, resulting in system errors for *R_rs_* measurement and making them highly sensitive to solar elevation angle variations and prone to sun glint. Additionally, the spectral precision and accuracy of hyperspectral imagers themselves may be inferior to dedicated spectrometers due to sensor noise, detector dark current, and spatial mixing pixel effects.

In light of these challenges, this study develops a UAV-based *R_rs_* measurement system (UAV-RRS) and evaluates its spectral accuracy. The system is designed to enable continuous and high-precision spectral observation of small-scale water bodies. Furthermore, we assess the consistency between UAV-derived reflectance and satellite-based atmospheric correction products. The integrated approach not only advances methodological frameworks for aquatic spectral monitoring but also provides critical insights into synergistic applications of multi-platform remote-sensing technologies.

## 2. Methods and Materials

### 2.1. Water R_rs_ Measurement Principle

The fundamental principle of above-water *R_rs_* measurement is based on the following equation [38,39]:

(1) $$\;\begin{array}{c} L_{sw}=L_{w}+r\cdot L_{sky}+L_{wc}+L_{g} \end{array}$$

where $L_{sw}$ is the total signal received by the detector, and $L_{w}$ represents the water-leaving radiance, which is the portion of light that enters the water, is scattered within, and exits the water surface back into the sensor. The term $r\cdot L_{sky}$ accounts for the sky radiance reflected directly off the water surface into the sensor, containing no inherent water body signal. Here, r is the air–water interface reflectance, typically derived empirically and with a mean value ranging between 0.022 and 0.028. Additionally, $L_{wc}$ represents the contribution from whitecaps on the water surface, while $L_{g}$ corresponds to the random reflection of direct solar irradiance caused by surface waves—neither of which contain any water body information. For field measurements, atmospheric scattering signals can generally be neglected. To obtain the water-leaving radiance $L_{w}$, the sky-glint component $r\cdot L_{sky}$ is subtracted from the total received signal $L_{sw}$.

To eliminate the effects of solar zenith angle, sensor characteristics, and other environmental factors, it is necessary to account for the prevailing illumination conditions. Therefore, the remote-sensing reflectance $R_{rs}$, which normalizes the water-leaving radiance by the incident irradiance, is computed using the following equation:

(2) $$\begin{array}{c} R_{rs}=\frac{L_{w}}{E_{d}\left(0^{+}\right)}=\frac{L_{sw}-r\cdot \;L_{sky}}{E_{d}\left(0^{+}\right)} \end{array}$$

where $E_{d}\left(0^{+}\right)$ represents the total downward irradiance incident on the water surface.

Due to the non-Lambertian characteristics of the water surface, it is necessary to establish an appropriate observation geometry. Traditional ground-based fixed-point in situ measurements commonly employ the ASD FieldSpec spectroradiometer (Malvern Panalytical Company, Longmont, CO, USA). The standard measurement protocol is as follows: The operator faces away from the sun, with the instrument’s observation plane forming an angle of 135° relative to the solar incident plane and an angle of 40° with the water surface normal. The total signal entering the sensor from the water surface is first measured. Then, the sensor is flipped while maintaining the same angle to measure the sky-diffused radiance. Finally, the sensor is directed at a horizontally placed, rigorously calibrated standard diffuse reflectance plate to measure the upwelling radiance, enabling the derivation of the total irradiance.

### 2.2. Architecture

**Figure 1:**
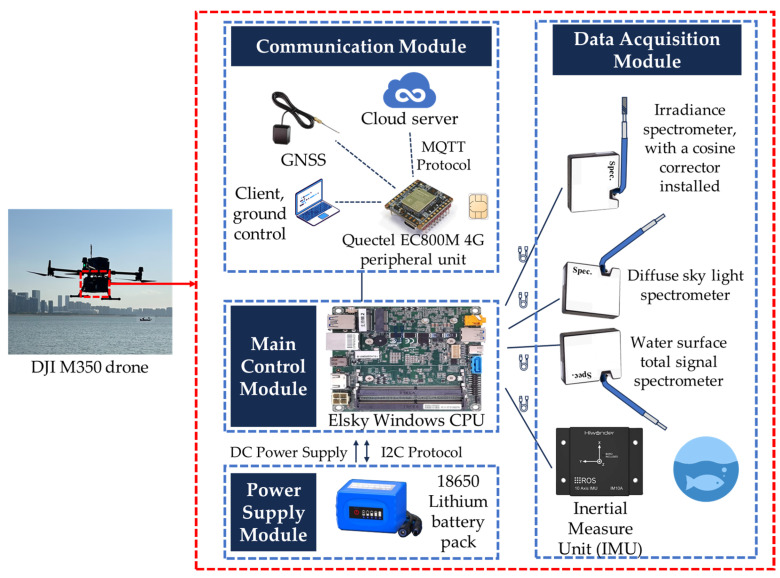
UAV-RRS architecture.

To achieve short-term, large-area monitoring of fine-scale watersheds, we developed a UAV-based hyperspectral *R_rs_* measurement system (UAV-RRS) based on the aforementioned principles, as illustrated in Figure 1. The system is mounted on a DJI M350 UAV (SZ DJI Technology Co., Ltd., Shenzhen, China) and consists of four main components: the control module, the data acquisition module, the communication module, and the power supply module.

The control module is based on the Elsky NUC700-UA CPU, featuring an i7-7660U processor, running on the Windows 10 operating system. With a power consumption of 15 W and 4 GB of memory, it ensures sufficient performance while maintaining low power usage. The entire system is powered by an 18650-lithium battery pack, providing a maximum endurance of up to 4 h. Additionally, it supports battery status monitoring via the I2C protocol, enabling a low-power warning function.

The data acquisition module primarily consists of three lightweight optical fiber spectrometers, all of which are Spectral Nexus GCS300 models (Futergy Marine Technology Co., Ltd., Hangzhou, China). Each unit weighs 700 g and features a crossed C-T optical path structure. The spectral range covers both visible and near-infrared bands, with a maximum resolution of 0.4 nm. It provides integration times starting at the 10 μs level and accepts fiber optic input via an SMA905 interface. More detailed parameters are shown in Supplementary file S1. The three spectrometers correspond to three measurement processes in ground-based in situ measurements: one measures irradiance vertically upwards, another measures sky scattered light at a 40° angle above the horizon, and the third measures the total water surface signal at a 40° angle below the horizontal. To address the small field of view of the optical fiber probe, the irradiance sensor is equipped with a cosine corrector at the fiber tip. This ensures the light field distribution within the upper hemisphere is homogenized and transformed into incident light signals according to Lambert’s cosine law. To accommodate the highly dynamic motion characteristics of the UAV platform, an inertial measurement unit (IMU) has also been integrated, allowing real-time monitoring of the platform’s attitude and velocity changes. These IMU data are independent of the UAV’s built-in IMU to prevent the system drifting along the time axis, providing auxiliary indicators for data quality control.

The communication module integrates a Quectel EC800M 4G peripheral unit, which transmits on-board data in real time to the ground station via 4G signals, while simultaneously backing up the data to a cloud platform. This enables remote monitoring and data networking. Additionally, the unit can also function as a GNSS module for sending and receiving data, using AT commands to obtain the current latitude and longitude coordinates. Like the IMU, the GNSS data obtained from the external device have a higher real-time match with the spectral data, as they are synchronized with the CPU clock. The error is minimized to a millisecond level, providing precise geospatial data integration.

A high-strength nylon shell was custom-designed using 3D printing. To ensure system load balance, the components are evenly distributed, with the entire system weighing 2.4 kg, which meets the payload requirements of the DJI M350 UAV.

### 2.3. Flight Parameters and Technical Configuration

To facilitate the accurate retrieval of *R_rs_*, UAV-RRS was employed and the flight and sensor configuration parameters are summarized below.

Flights were executed autonomously following a predefined trace aligned with river geometries. The typical flight altitude ranged between 5 and 20 m above the water surface due to the spectral instrument’s detection range limitations, with a cruising speed of approximately 5 m/s. Given the low flight altitude, manual flight mode was employed to mitigate potential risk factors. Data were collected under stable lighting conditions—between 9:00 and 15:00 local time—on cloud-free days to minimize atmospheric variability. The overall endurance time is influenced by local temperature and wind speed. Testing shows that under most suitable flight conditions, a single UAV battery supports a flight time of 20 to 30 min. At a flight speed of 5 m/s, it can cover a watershed length of 6 to 9 km. The spatial accuracy of the measurements depends on the UAV’s flight speed and the sampling time per data point. With a balance of spatial accuracy, spectral response signal-to-noise ratio (SNR), and system memory, a typical sampling frequency of 0.5–1 s is used, ensuring a resolution of less than 10 m even at high flight speeds. This meets the requirements for quantitative water remote sensing. During data collection, the ADC method is employed to resample the data, storing them as u16 integers and encapsulating them into frames for transmission to the ground and cloud platforms, reducing memory pressure on both the system and the network.

To facilitate the accurate retrieval of *R_rs_*, UAV-RRS was deployed with specific flight and operational parameters as summarized in Table 1. This configuration was optimized for reliable spectral acquisition over water bodies.

### 2.4. Dark Current Correction

The correct use of the instrument primarily relies on the precise correction and calibration of the lightweight spectrometers. Due to the presence of a charge-coupled device (CCD) dark current, each measurement is subject to background noise, necessitating dark current correction. The primary cause of the dark current is that CCDs are typically made from semiconductor materials, such as silicon, and at room temperature, thermal energy excites electrons from the valence band to the conduction band, generating thermal noise. Additionally, impurities and crystal defects inside the CCD can create trap levels, which increase the probability of thermal excitation of charge carriers. The MOSFETs (e.g., output amplifiers) and other components in the CCD’s readout circuitry may also contribute to small leakage currents, which accumulate as stable offset noise, further affecting the dark current.

The spectrometer’s actual DN reception range is from 0 to 65,535. Dark current measurements were taken under different integration times to examine its stability. Considering the highly dynamic flight conditions of the field UAV platform and the generally sufficient and stable lighting conditions during the actual experiments, the integration time of spectrometers is typically kept short, usually within 20 ms. Therefore, during dark current correction, the dark current noise was measured under 20 sets of integration times ranging from 1 to 20 ms for three spectrometers, with five measurements taken per integration time.

The fiber optic cable of the spectrometer was covered with a black plastic sleeve to ensure complete light blocking during the measurements, and a 60 min preheating period was used to stabilize the temperature.

### 2.5. Radiometric Calibration

To achieve quantitative monitoring of water body radiation, it is essential to perform the strict calibration of each spectral device [40]. The calibration of the spectrometers includes wavelength calibration and radiometric calibration.

Wavelength calibration ensures that the wavelength axis of the spectrometer matches the true spectral wavelengths and corrects for wavelength drift. This calibration is typically performed using a known-wavelength standard light source (e.g., mercury lamps, neon lamps, xenon lamps, etc.), by measuring the characteristic spectral lines of the emitted light and matching the pixel positions measured by the spectrometer with the standard wavelengths, followed by polynomial regression fitting [41]. In our system, the spectrometers used have been pre-calibrated by the manufacturer, and measurements indicate that the wavelength error compared to the standard light source center wavelength is less than 1 nm.

Radiometric calibration is used to convert the raw DN radiance values measured by the spectrometer into physical radiometric quantities (such as radiance or irradiance), ensuring the comparability of data under different measurement conditions. The commonly used methods for radiometric calibration include laboratory integrating sphere calibration [42], outdoor diffuse reflectance calibration [43], and on-orbit calibration [44,45]. Due to the susceptibility of fiber optic spectrometers to environmental factors such as temperature variations, calibration is required before each experiment. Thus, the outdoor diffuse reflectance calibration method is more practical. Therefore, we chose this method to perform radiometric calibration on the three lightweight spectrometers used in our experiments.

The three lightweight spectrometers, as described in Section 2.2, have their optical fibers fixed to the spectrometer interfaces and are all integrated into the whole system. The other equipment used for calibration includes a diffuse reflectance plate with known reflectance values and the strictly radiometrically calibrated ASD FieldSpec 4 Hi-Res. The ASD spectrometer measures spectral ranges from 350 to 2500 nm, with a resolution of 3 nm and 8 nm in different wavelength ranges. The diffuse reflectance plate was calibrated for reflectance in a standard darkroom, with a range from 400 to 900 nm. The experiment was conducted on a clear, sunny day with no clouds, in an outdoor, open, unobstructed area.

**Figure 2:**
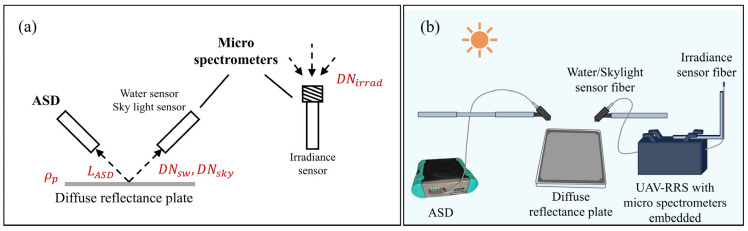
Micro spectrometer’s radiometric calibration using diffuse reflectance plate. (**a**) Calibration process illustration. (**b**) Demonstration of experimental equipment.

The fiber probe of the ASD spectrometer and the fiber probes of the lightweight spectrometers (water surface and sky light spectrometers) were aligned with the central area of the diffuse reflectance plate (for the irradiance spectrometer, the probe should be oriented vertically upwards to directly receive the irradiance signal from the upper hemisphere). The upward radiance measured by the ASD and the DN radiance values measured by each spectrometer were recorded. The overall experimental schematic and instrument setup illustrations are shown in Figure 2. The field radiance calibration formula for the water surface and sky light sensors is given by Equation (3), and the field irradiance calibration formula for the irradiance sensor is given by Equation (4).

(3) $$\begin{array}{c} L_{ASD}\left(\lambda \right)=DN_{i}\left(\lambda \right)\times ratio_{i}\left(\lambda \right)\times ITG+DC_{i}\left(\lambda \right),\;i=1,\;2 \end{array}$$

(4) $$\begin{array}{c} E_{s}\left(\lambda \right)=\frac{\pi \cdot L_{ASD}\left(\lambda \right)}{\rho_{p}\left(\lambda \right)}=DN\left(\lambda \right)\times ratio\left(\lambda \right)\times ITG+DC\left(\lambda \right) \end{array}$$

In Equation (3), $L_{ASD}\left(\lambda \right)$ represents the upward radiance measured by the ASD after reflection from the diffuse reflectance plate. Due to the Lambertian characteristic of the diffuse reflectance plate, this value is identical to the radiance received by the lightweight spectrometer. $DN_{i}\left(\lambda \right)$ refers to the DN value measured by the water surface or sky light spectrometer when aligned with the diffuse reflectance plate at the same time. ITG denotes the integration time of the lightweight spectrometer at the current moment (in millisecond), and $DC_{i}\left(\lambda \right)$ represents the dark current at the current integration time. From this, the radiometric calibration coefficient $ratio_{i}\left(\lambda \right)$ can be determined. It represents the radiance of the ambient light field corresponding to the DN value measured by the lightweight spectrometer per unit time after dark current correction.

Equation (4) follows a similar conversion process as Equation (3), with the main difference being that Equation (4) refers to the conversion process for the irradiance spectrometer. Since this spectrometer directly senses irradiance vertically upwards, the radiance measured by the ASD from the diffuse reflectance plate needs to be further converted to the corresponding irradiance $E_{s}(\lambda )$. In this equation, $\rho_{p}\left(\lambda \right)$ represents the reflectance of the diffuse reflectance plate at each wavelength.

We conducted measurements from 9:30 A.M. to 3:00 P.M., with measurements every half hour, totaling 12 time points. This allowed us to test the stability of the conversion coefficients under different light field conditions.

Since the spectral bands of the ASD and the lightweight spectrometers are different, cubic spline interpolation was used to resample spectral bands to the same wavelength range with a 1 nm resolution. Given that the spectral resolutions of all instruments are high, the errors introduced by resampling are smaller than the random signal errors, and therefore, have a negligible impact on the results. A detailed comparison between resampling error and random signal error can be found in Appendix A. The final calibration results, represented by the radiometric calibration coefficients, are quantified using statistical metrics such as the standard deviation (SD), root mean squared error (RMSE), and coefficient of variation (CV) to assess the differences among the measurements. The formulas for these metrics are as follows:

(5) $$\begin{array}{c} {SD}_{\lambda }=\sqrt{\frac{1}{N}\sum_{i=1}^{N}{\left(S_{i,\lambda }-{\bar{S}}_{\lambda }\right)}^{2}} \end{array}$$

(6) $$\begin{array}{c} RMSE=\sqrt{\frac{1}{N}\sum_{i=1}^{N}{\left(S_{i,\lambda }-{\bar{S}}_{\lambda }\right)}^{2}} \end{array}$$

(7) $$\begin{array}{c} CV=\frac{{SD}_{\lambda }}{{\bar{S}}_{\lambda }} \end{array}$$

where $S_{i,\lambda }$ is the spectral calibration result curve from each measurement, ${\bar{S}}_{\lambda }$ is the average spectral calibration curve, and N is the total number of measurements taken throughout the day.

### 2.6. Experiments of In Situ–UAV–Satellite Joint Comparison

#### 2.6.1. Experiment Area

**Figure 3:**
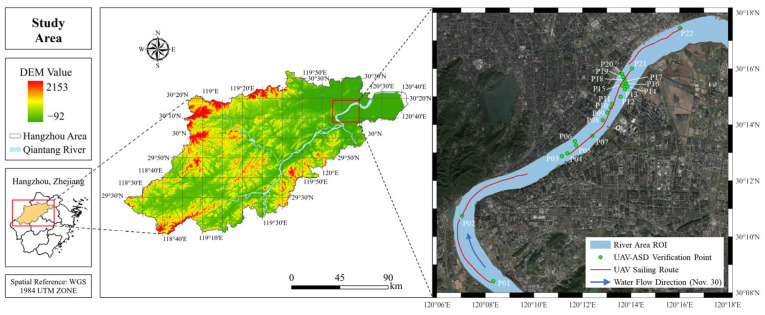
Experiment area and locations of sampling points.

To evaluate the performance of UAV-RRS for fine-scale inland water body *R_rs_* monitoring, we conducted field experiments. This study selected the middle reaches of Qiantang River in Hangzhou city (120°06′–120°18′ E, 30°08′–30°18′ N) as the target water area (Figure 3). Qiantang River is located in the subtropical monsoon climate zone, with an annual average precipitation of about 1450 mm, and is significantly influenced by both tides and runoff [46,47]. The upstream Fuchun River Reservoir regulates the outflow, while the downstream experiences tidal influences from Hangzhou Bay, forming a unique “semi-diurnal” hydrological dynamic. The concentration of TSM exhibits significant spatial and temporal variability. The river width ranges from 500 to 1200 m, with an average depth of 4 to 8 m and flow velocity ranging from 0.5 to 2.5 m/s (up to 3.5 m/s during ebb tide). The coastal landform is mainly composed of alluvial plains, with localized river sandbars and shallow wetland areas. The river’s meandering coefficient ranges from 1.2 to 1.5, creating a diverse water body reflectance spectrum background.

The experiment area is in the middle section of Qiantang River. The total length of the river channel is approximately 30 km. This section represents a typical river reach in the Qiantang River basin, characterized by the highest level of urbanization, complex hydrological conditions, and a sensitive ecological environment.

#### 2.6.2. Data Acquisition

To verify the instrument accuracy, we conducted experiments from 27 November to 30 November in 2024, in the autumn season, over a period of four days in the aforementioned study area. During the experiment, it remained a relatively stable environment with minimal cloud coverage. Wind speeds were lower than 4 m/s with weak surface waves. On-site data were measured using the ASD FieldSpec 4 ground spectrometer via the above-water method. We sailed along the river aboard an inland hydrological survey boat and measured the water surface *R_rs_* spectra at 22 locations (P01–P22), as shown in Figure 3. After completing the ASD measurements, the airborne hyperspectral equipment was activated, with the spectral device set to continuous measurement mode. The UAV took off from the boat’s open deck, reaching an altitude of about 5 m and a distance of approximately 10 m from the boat to minimize the impact of the vessel on spectral measurements, and it hovered for one minute before returning and landing.

**Figure 4:**
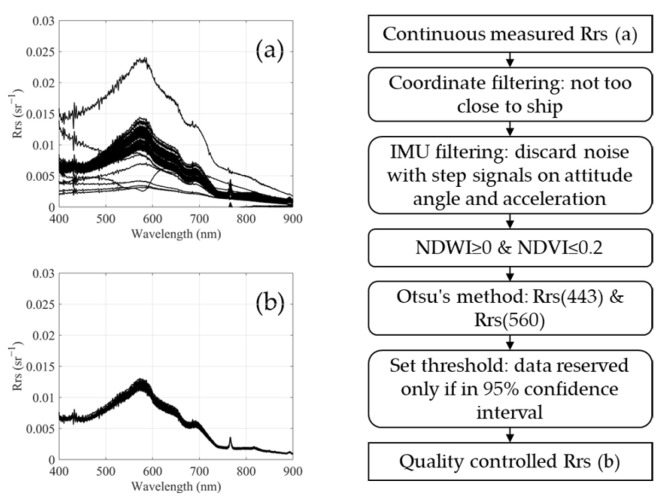
Procedure of data quality control. (**a**) Continuously measured *R_rs_* before processing. (**b**) Quality-controlled *R_rs_* result.

Both the ASD and UAV-RRS were positioned very close to the water surface during measurements, allowing for the neglect of atmospheric influences and thus avoiding complex atmospheric correction processes. This made the two datasets directly comparable. For the UAV, however, since it continuously measured data from takeoff to landing, there were numerous redundant erroneous data points that required quality control. Multiple steps, including geographic range limitations, IMU posture and acceleration filtering, NDWI and NDVI index screening, and spectral clustering for noise reduction, were applied to obtain accurate spectral results, as shown in Figure 4. The quality-controlled spectral data were then averaged and compared with the on-site ASD data collected from the boat.

To demonstrate the advantages of water spectral monitoring using the UAV platform, we conducted a continuous flight experiment along the entire river section on the final day. To save time and measure the longest possible continuous UAV spectrum, we did not measure the ASD spectrum on this day. The experiment lasted from 9:30 A.M. to 2:30 P.M., involving multiple flight sorties. The full flight trajectory is shown as the red line within the basin in Figure 3.

We selected the Sentinel-2A satellite as the reference source for satellite remote sensing comparison. Sentinel-2 is a high-resolution multispectral remote-sensing satellite under the European Space Agency’s (ESA) Copernicus program, primarily designed for land and coastal water monitoring. The series includes Sentinel-2A, launched in 2015, and Sentinel-2B, launched in 2017, working together to achieve a global coverage cycle of five days. Sentinel-2 is equipped with a 13-band multispectral instrument (MSI), covering visible, near-infrared (NIR), and shortwave infrared (SWIR) wavelengths, with spatial resolutions ranging from 10 m (visible and NIR) to 60 m (atmospheric correction bands). Its data are widely used in vegetation monitoring, water environment assessment, crop growth evaluation, and land-cover classification. Specifically, Sentinel-2 is highly suitable for water remote-sensing applications, including water color monitoring, water quality parameter retrieval, and water transparency assessment.

In this study, we selected Sentinel-2A MSI Level-1C (L1C) imagery acquired during the field campaign, specifically the scene passing over the study area on 28 November at 10:35 a.m. local time. This dataset provides the top-of-atmosphere (TOA) reflectance and was downloaded from the ESA Copernicus Space Data Ecosystem (CDSE) Portal (https://dataspace.copernicus.eu/, accessed on 30 November 2024). The selected scene, located on 51RTP mosaic, corresponds to orbit 089 and processing baseline (PDGS) 0511, covering the study area with minimal cloud cover and high data quality.

To derive *R_rs_* over water, atmospheric correction was applied. Using ESA’s Sentinel Application Platform (SNAP), multi-band data were resampled to ensure a uniform spatial resolution across all bands. We tested multiple atmospheric correction algorithms, including C2RCC, FLAASH, 6S, Acolite, SWIR-AC, iCor, and Sen2Cor—seven widely used methods (detailed acquisition and implementation procedures are listed in Table 2). The corrected results were then compared with the UAV-RRS measurements.

After completing atmospheric correction, all output results were transformed from the UTM projection coordinate system to the geographic coordinate system to facilitate a more intuitive comparison with UAV-derived data. Notably, only the Acolite and SWIR-AC algorithms directly provide *R_rs_* values, while the remaining algorithms yield reflectance (BOA, bottom of atmosphere) as the output, which requires further conversion to *R_rs_*. The conversion is performed using the following equation:

(8) $$\begin{array}{c} R_{rs}=\frac{reflectance}{\pi } \end{array}$$

**Figure 5:**
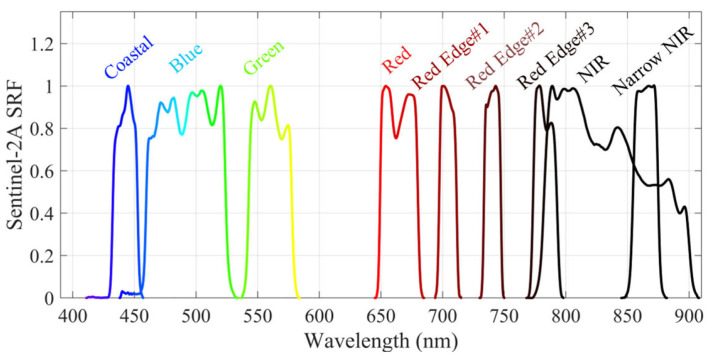
Spectral response function of Sentinel-2A, first 9 bands (B01-B8A).

Since the UAV acquires hyperspectral data with continuous spectral coverage, whereas Sentinel-2 provides discrete spectral bands, a spectral resampling process is required to facilitate a meaningful comparison between the two datasets. This is achieved using the spectral response function (SRF, see https://nwp-saf.eumetsat.int/site/software/rttov/download/coefficients/spectral-response-functions/, accessed on 29 April 2025). The hyperspectral data are transformed into discrete spectra corresponding to Sentinel-2 bands using the following equation:

(9) $$\begin{array}{c} {Rrs}_{band}=\frac{\int_{band_{\lambda \;min}}^{band_{\lambda \;max}}SRF_{band}\left(\lambda \right)Rrs\left(\lambda \right)d\lambda }{\int_{band_{\lambda \;min}}^{band_{\lambda \;max}}SRF_{band}\left(\lambda \right)d\lambda } \end{array}$$

where $Rrs\left(\lambda \right)$ represents the hyperspectral *R_rs_* of the UAV at wavelength λ. The final transformed reflectance, $Rrs_{band}$, corresponds to the simulated Sentinel-2 spectral bands. Given that the UAV hyperspectral sensor operates within the 400–900 nm range, only the first nine Sentinel-2 bands (B01–B8A) are utilized for spectral resampling, as illustrated in Figure 5.

The evaluation of ASD in situ measurements against UAV-derived continuous spectra, as well as UAV spectra against Sentinel-2 atmospheric correction results, was conducted using the following statistical metrics [49,50,51,52]: Pearson correlation coefficient (PCC, r), root mean squared error (RMSE), mean absolute deviation (MAD), mean relative error (MRE), and structural similarity index (SSIM).

(10) $$\begin{array}{c} r=\frac{\Sigma \left(S_{1,i}-\bar{S_{1}}\right)\left(S_{2,i}-\bar{S_{2}}\right)}{\sqrt{\Sigma {\left(S_{1,i}-\bar{S_{1}}\right)}^{2}{\left(S_{2,i}-\bar{S_{2}}\right)}^{2}}} \end{array}$$

(11) $$\begin{array}{c} RMSE=\sqrt{\frac{1}{N}{\left(S_{1,i}-S_{2,i}\right)}^{2}} \end{array}$$

(12) $$\begin{array}{c} MAD=\frac{1}{N}\sum_{i=1}^{N}\left|S_{1,i}-S_{2,i}\right| \end{array}$$

(13) $$\begin{array}{c} MRE=\frac{1}{N}\sum_{i=1}^{N}\left|\frac{S_{1,i}-S_{2,i}}{S_{1,i}}\right| \end{array}$$

(14) $$\begin{array}{c} SSIM_{1,2}=\frac{\left(2\mu_{1}\mu_{2}+C_{1}\right)\left(2\sigma_{1,2}+C_{2}\right)}{\left(\mu_{1}^{2}+\mu_{2}^{2}+C_{1}\right)\left(\sigma_{1}^{2}+\sigma_{2}^{2}+C_{2}\right)} \end{array}$$

where the subscripts 1 and 2 denote the reference and comparison datasets, respectively, such as ASD in situ spectra versus UAV-derived continuous spectra, and UAV spectra versus satellite-derived atmospherically corrected spectra. $S_{i}$ represents the spectral value at a given band. Both $\bar{S}$ and μ denote the mean spectral value. $\sigma_{1}$ and $\sigma_{2}$ correspond to the variances of the two spectral datasets, while $\sigma_{1,2}$ represents their covariance. $C_{1}$ and $C_{2}$ are small constants (set to 0.001) introduced to prevent division by zero.

## 3. Results

### 3.1. Dark Current Correction Result

**Figure 6:**
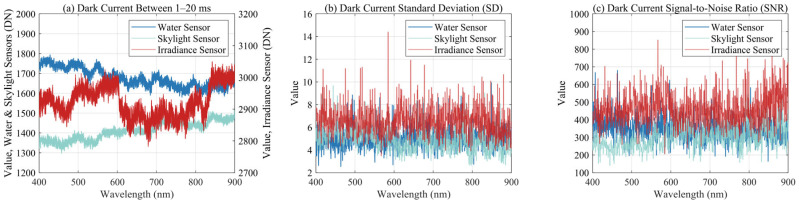
Dark current with integration time from 1 ms to 20 ms. (**a**) Dark current of all three spectrometer sensors. (**b**) Dark current standard deviation of three spectrometer sensors. (**c**) Dark current SNR of three spectrometer sensors.

The optical performance measurement and calibration of the spectrometers are crucial to the overall accuracy of the UAV-RRS system. We first measured the dark current effect of the instruments, and the dark current results within the range of 1–20 ms are shown in Figure 6.

Within the small integration time range, the effect of different integration times on dark current noise is not significant and is much smaller than the random errors (Figure 6a,b). The average standard deviation of the dark current is generally less than 10 DN, indicating a very small range. Further calculations of the dark current SNR show that most bands have an SNR between 200 and 700. For water bodies, international satellites such as MODIS Terra/Aqua, MERIS Envisat, VIIRS, and OLCI Sentinel-3A/B have SNRs greater than 1000 for ocean color measurements [53]. Meanwhile, SNRs for inland water monitoring from satellites like Landsat OLI and HJ-1 are greater than 100 [54,55]. Therefore, we consider an SNR greater than 100 to be suitable for inland water monitoring sensors.

In the actual process of water body *R_rs_* measurement, the SNR calculation formula should be the effective signal, excluding the dark current, divided by the noise signal. For measurement purposes, the standard deviation is typically used as a proxy for noise (Equation (15)) [56]. Therefore, the effective signal measured by the three spectrometers should have a DN value of at least 1000.

(15) $$\begin{array}{c} SNR=\frac{DN_{total}-DN_{darkcurrent}}{DN_{noise}} \end{array}$$

During measurements, the integration time is typically set based on the ambient light conditions, ensuring that the DN signal falls within the 40–80% acceptance range (26,000–52,000 DN) to be robust enough. According to multiple field measurements conducted under clear sky conditions, the integration time of the sky light diffuse reflectance spectrometer is generally between 1 and 3 ms to ensure signal levels within the optimal DN range. Due to the weaker signal reflected from the water surface, the integration time of the spectrometer is typically around 5 ms. For the irradiance spectrometer, which is equipped with a cosine corrector, the light is corrected according to the cosine law for the upper hemisphere, resulting in significantly weaker intensity compared to the bare fiber optic. Therefore, an integration time of around 10 ms is generally considered reasonable.

### 3.2. Radiometric Calibration Result

**Figure 7:**
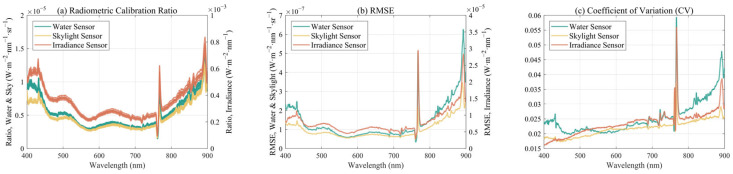
Radiometric calibration results all day long. (**a**) Radiometric calibration ratio curve of three spectrometer sensors. (**b**) RMSE of ratio results. (**c**) CV of ratio results.

Radiometric calibration converts the DN signal values measured by the spectrometer into the actual effective radiative signal of the ambient light field. The results of the radiometric calibration experiment throughout the day are shown in Figure 7. The irradiance spectrometer measures the ambient light field irradiance, while the water surface spectrometer and sky light spectrometer measure the radiance reflected from the standard diffuse reflectance plate, which can also be converted into irradiance. As a result, the measured DN curves are similar, and the resulting calibration coefficient curves also exhibit similar shapes (Figure 7a), generally showing a trend of low values in the middle and high values at both ends. This pattern aligns with the characteristic of solar radiation being concentrated in the visible light region.

The RMSE and CV results (Figure 7b,c) demonstrate that the measurement errors are orders of magnitude smaller than the measured signal, indicating exceptionally high calibration accuracy.

From the actual results of the calibration curve, the standard deviation is very small. The visible light bands show good aggregation, while some bands in the ultraviolet and infrared regions are slightly worse. However, overall, all meet the accuracy requirement of below 4%, indicating that the equipment has good reliability.

### 3.3. In Situ–UAV Comparison

**Figure 8:**
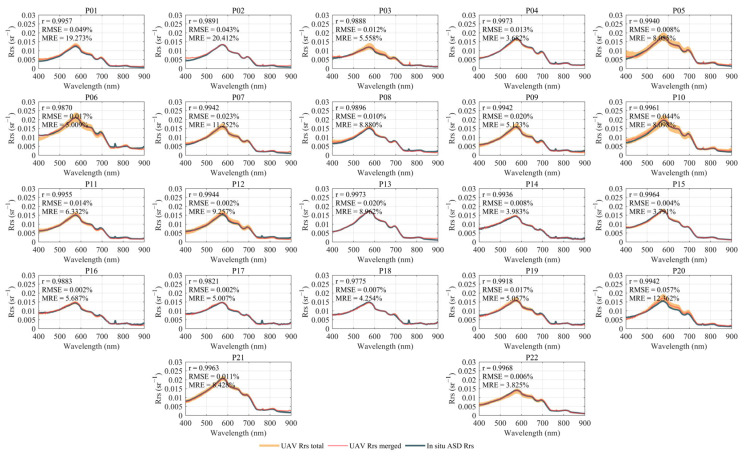
In situ ASD- and UAV-measured *R_rs_* in 22 stations.

**Figure 9:**
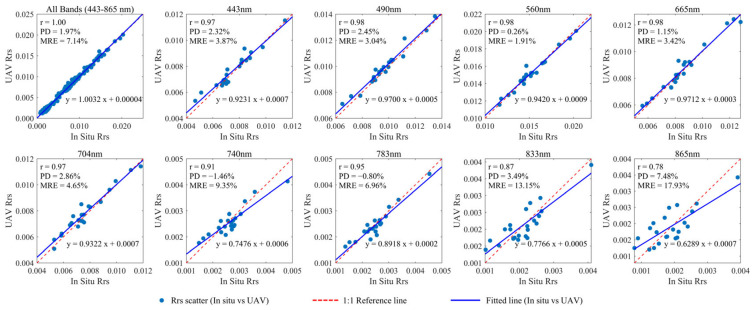
In situ ASD and UAV waveband-specific measured *R_rs_* 1:1 spectral comparison.

We present a comparison of the *R_rs_* spectra measured by the ASD boat-based method and UAV-RRS (Figure 8), and select several bands for 1:1 comparison (Figure 9). Figure 8 presents the in situ *R_rs_* spectra measured by ASD at all 22 sites, the full set of UAV-RRS spectra collected during the hovering measurement stage (denoted as UAV Rrs total), and the averaged UAV-RRS spectrum for each site (denoted as UAV Rrs merged). The majority of the *R_rs_* measurements from the UAV platform show excellent agreement with the in situ ASD boat-based measurements. The measurement deviations of UAV-RRS for single points are also relatively small. In the selected study area, the optical properties of the water body exhibit minimal variation, with the overall shape remaining consistent. Only slight differences are observed in the peak values and local band intensities.

The Qiantang River estuary is characterized by high turbidity due to suspended sediment, which corresponds to higher values in the infrared spectrum. Conversely, the upstream water is clear, representing typical clean water. From the spectral characteristics of the water body in the study area, most of the high *R_rs_* is concentrated in the visible light range, with extremely weak infrared signals. The ASD-UAV *R_rs_* comparison by bands also shows that, for clean water bodies, the UAV spectral measurements in the visible light range closely match the true values, with a correlation coefficient above 0.9. However, in the infrared range, the signals are very weak, and the accuracy is slightly lower compared to the visible light bands.

**Figure 10:**
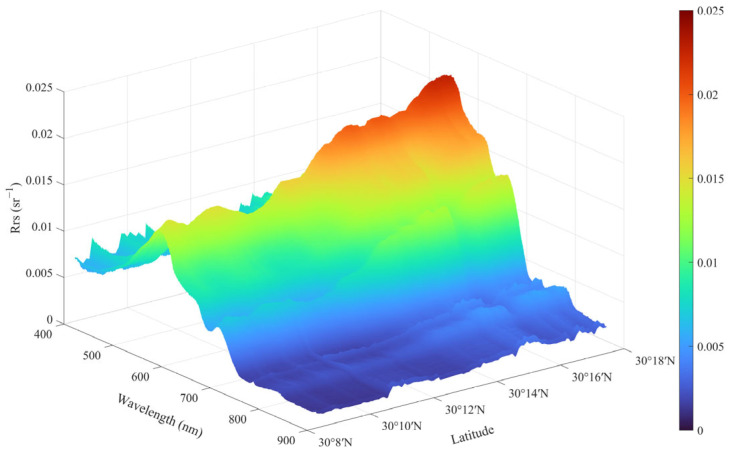
River-long UAV-RRS measured *R_rs_* spectrum.

To show the spatial distribution of the water body spectra in the study area’s watershed segment, we plotted the distribution of the continuous spectra along the latitudinal direction (Figure 10), which roughly corresponds to the actual northeast–southwest orientation. These continuous spectra were acquired during UAV flights conducted throughout the day on 30 November 2024. Based on the UAV’s full flight spectral data, the data were resampled according to a preset grid network (0.001° × 0.001°, WGS84 coordinate system), and a two-dimensional cubic spline interpolation method was applied to fill the entire region of interest area.

**Figure 11:**
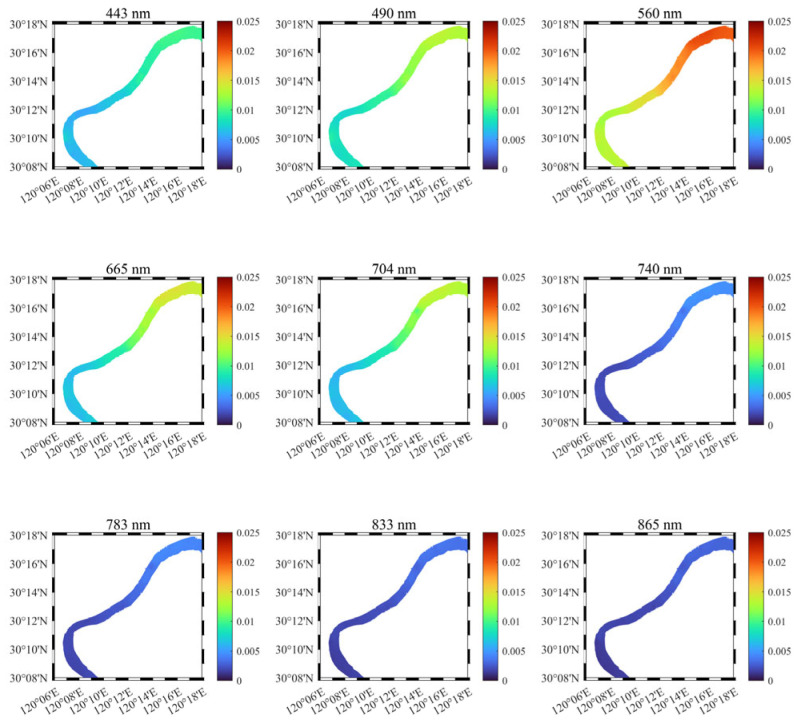
UAV-RRS continuously measured *R_rs_* by waveband.

The results for key bands are shown in Figure 11. It can be observed that the *R_rs_* spectral peaks are relatively low in the upstream region, around 0.01–0.015 ${\mathrm{sr}}^{-1}$, while the downstream region reaches 0.02–0.025 ${\mathrm{sr}}^{-1}$. The shape of the spectra remains largely unchanged, indicating that the relative content of the water body’s components remains consistent, with the composition of the upstream and downstream water bodies being stable. Similarly, it can be noted that starting from the infrared bands, the *R_rs_* spectra are mostly below 0.005 ${\mathrm{sr}}^{-1}$, with extremely weak signals.

### 3.4. UAV–Satellite Comparison

**Figure 12:**
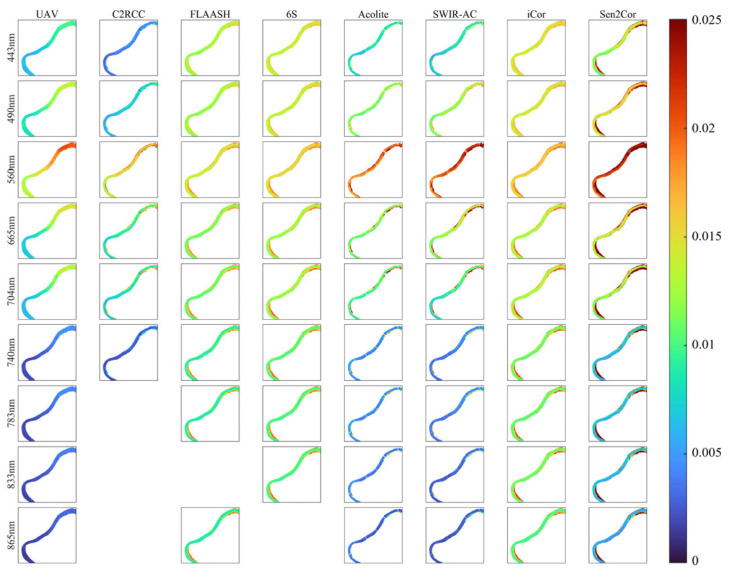
Comparison of UAV-RRS and Sentinel-2 atmospheric corrected *R_rs_* at different bands.

To demonstrate the differences in precise quantitative remote sensing of small water bodies between the UAV and satellites, we compared the UAV results with those obtained using seven common atmospheric correction algorithms, as shown in Figure 12. In the results, the C2RCC algorithm only provides results for the first six bands, the FLAASH algorithm is missing the B08 band (833 nm), and the 6S algorithm lacks the B8A band (865 nm), while the other algorithms provide complete results for the first nine bands.

The atmospheric correction results for all algorithms show a similar basic trend across the bands, gradually increasing from the ultraviolet to the visible bands, peaking at B03 (560 nm, green), and then decreasing steadily from B04 (665 nm, red) through the infrared bands. However, the trends in the algorithms vary significantly. The FLAASH, 6S, and iCor algorithms show little variation across the ultraviolet-visible-infrared spectrum, while the C2RCC, Acolite, SWIR-AC, and Sen2Cor algorithms more closely match the magnitude of changes observed in UAV measurements.

Spatial comparisons between the UAV and the various algorithms reveal that the UAV measurements exhibit more pronounced spatial heterogeneity, with significant peak value differences between the upstream and downstream regions. The values generally follow a two-segment distribution pattern, with a step-like phenomenon at the center of the study area. In contrast, the atmospheric correction algorithm results from the satellite tend to be more spatially uniform, with less pronounced upstream–downstream differences compared to the UAV.

**Figure 13:**
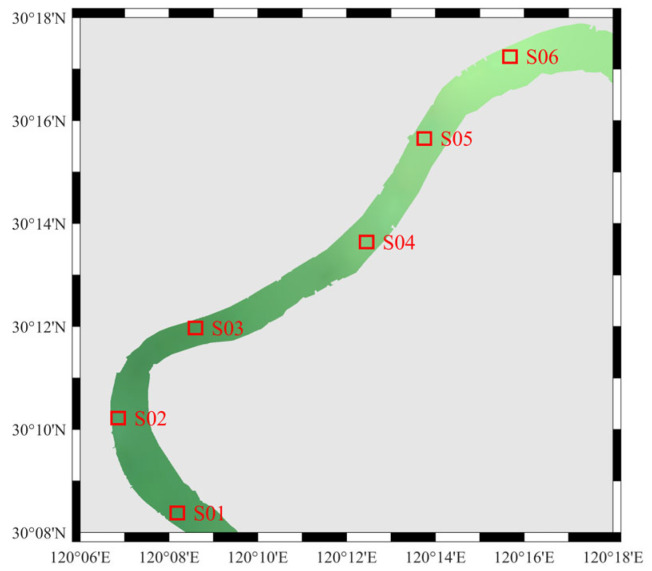
Selected comparison sites S01–S06 for UAV and Sentinel-2 atmospheric corrected *R_rs_*.

**Figure 14:**
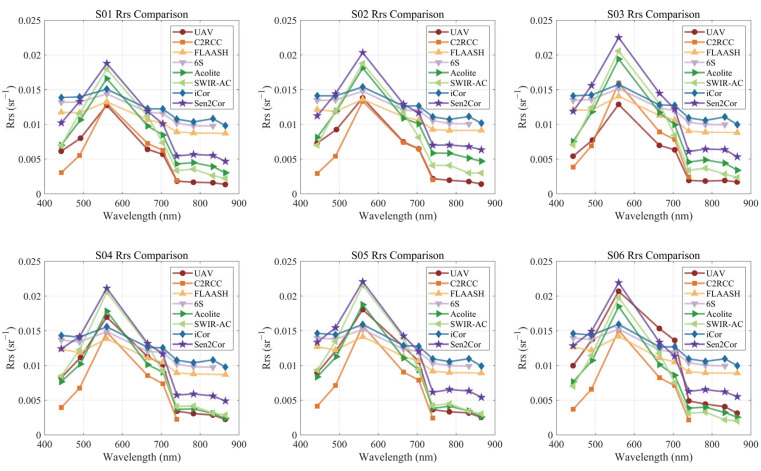
Comparison of UAV and Sentinel-2 atmospheric corrected *R_rs_* at selected S=stations.

To visually compare the spectral differences between the UAV and various satellite atmospheric correction algorithms, six points were selected from the study area. These points were evenly spaced from upstream to downstream and essentially cover the entire research area, named S01 to S06, as shown in Figure 13. The spectral curves for each point are shown in Figure 14. The previously mentioned conclusions are even more evident in Figure 14. The FLAASH, 6S, and iCor spectral curves are smoother compared to the UAV and the other algorithms, showing certain similarities in trends, but differing significantly in absolute values. In contrast, the other algorithms more closely match the UAV results.

Nevertheless, among the six stations from upstream to downstream, the UAV shows the most significant increase in peak values. At 560 nm, the UAV value at S01 is nearly equal to the lowest value of the atmospheric correction algorithms, while at S06, the UAV value at 560 nm is close to the highest value observed among all the atmospheric correction algorithm results. Although the atmospheric correction algorithms also show a certain trend of increasing spectral peak values downstream, this trend is less pronounced compared to the UAV results. This suggests that the fine-scale satellite-based quantitative remote sensing for small watersheds has a degree of underfitting and cannot effectively capture the internal differences.

**Figure 15:**
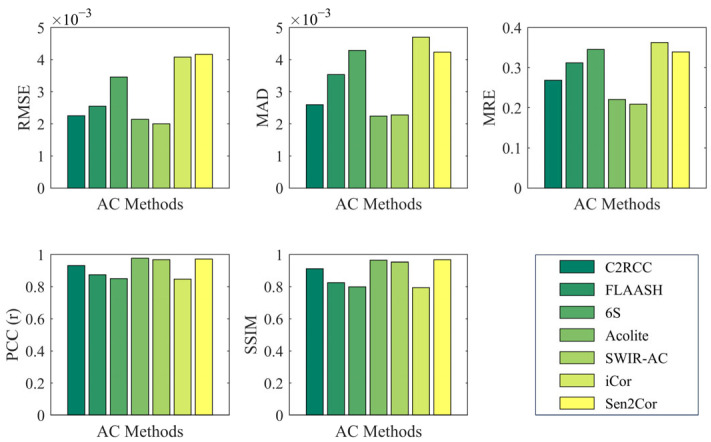
Statistical result of Sentinel-2 atmospheric corrected *R_rs_* compared to UAV.

We integrated the full UAV data and the corresponding satellite atmospheric correction algorithm results, using UAV data as the ground truth, to evaluate the accuracy of each atmospheric correction algorithm. The statistical results are shown in Figure 15. It should be noted that since C2RCC only includes the first six bands and the *R_rs_* signal in the infrared part of the study area is weak, the statistical results are all based on the average statistics of the first six bands.

Among the evaluation metrics, RMSE, MAD, and MRE were used to measure the errors between the satellite atmospheric correction algorithm results and the UAV data. The three algorithms with relatively smooth spectra—FLAASH, 6S, and iCor—showed relatively large errors. Additionally, although C2RCC appears similar to UAV in spectral results, it tends to be biased high overall, resulting in larger relative errors. Among the remaining three algorithms, Acolite and SWIR-AC exhibited the smallest relative errors, with C2RCC slightly worse than these two.

The PCC and SSIM metrics were used to assess the similarity of spectral curves. The spectral trends of the seven algorithms were generally consistent with UAV, so both PCC and SSIM were above 0.8. Similarly, FLAASH, 6S, and iCor, which had smooth spectral variations, performed worse than the other algorithms in terms of similarity. Although the Sen2Cor algorithm showed a significant deviation in absolute values, its spectral shape was highly similar to UAV, with a similarity above 0.95, comparable to Acolite and SWIR-AC, and slightly better than C2RCC.

Considering both relative errors and spectral similarity results, Acolite and SWIR-AC showed the best agreement with UAV ground truth in terms of both spectral values and shape. C2RCC performed slightly worse, while Sen2Cor, despite larger numerical differences, had a very similar shape to UAV. The remaining algorithms—FLAASH, 6S, and iCor—showed a degraded performance due to their smooth spectral curves, exhibiting both numerical differences and shape discrepancies compared to the other algorithms.

## 4. Discussion

### 4.1. Assessment of In Situ–UAV Comparison

The close agreement between in situ ASD boat-based and UAV-RRS measurements validates the effectiveness of our UAV-based approach. At all discrete comparison sites, the correlation between the in situ and UAV-RRS spectral signals was very high (r > 0.9), which also demonstrates that the outdoor diffuse reflectance calibration method we employed is not only easy to operate but also ensures that the sensors maintain a very high spectral accuracy during field measurements.

In the near-infrared region (800–900 nm), the consistency between in situ measurements and UAV measurements is slightly lower. The main reason for this is the strong absorption of water in these bands, resulting in low signal-to-noise ratios. The inherently weak *R_rs_* signals (<0.005 sr⁻^1^) amplify the relative impact of measurement uncertainty. Considering that ASD spectral measurements are subject to fluctuations due to factors such as boat movement and bidirectional angle errors, as well as the very low *R_rs_* values, the infrared results can still be considered to closely match.

Compared to the ASD discrete measurement points with a larger time span, the continuous spectral measurements with UAVs along the river revealed a clear spatial pattern with higher *R_rs_* values downstream. This pattern, which might be missed by discrete sampling methods, highlights a key advantage of the UAV-RRS system: its ability to capture spatial heterogeneity in water optical properties at scales relevant to small water bodies.

### 4.2. Analysis of UAV–Satellite Comparison

Compared to the high agreement between UAV and ground-based in situ *R_rs_*, the comparison results between various atmospheric correction algorithms for Sentinel-2 and UAV-RRS exhibit greater discrepancies. Although the optical properties of the water body suggest that the water in the study area is more influenced by the upstream clear water, indicating that the area remained largely stable during the experiment in a period of low tidal conditions. However, the two-day time window and the continuous UAV flight, with measurements taken at different times of the day, still resulted in spectral differences between the UAV and satellite imagery within the experiment area. This also highlights the need for higher temporal frequency and more detailed observations of inland water bodies if necessary.

At the same time, while modern atmospheric correction algorithms have significantly improved, they continue to face challenges due to low signal-to-noise ratios over water bodies, especially in inland and coastal environments, where aerosol properties can vary considerably. For example, FLAASH and iCor are both based on MODTRAN radiative transfer model, and 6S is a physics-based algorithm using vector radiative transfer theory [57,58]. The three algorithms are all widely used for land applications. Although they are adaptable to water with appropriate parameterization, they are all tend to produce smoother spectral results due to its generalized approach. In addition, the need for extensive and detailed atmospheric characterization inputs means that coarse parameter inputs can significantly impact and reduce the accuracy of the model. In contrast, Acolite and SWIR-AC are specifically developed for aquatic applications [59,60]. They both assume negligible water-leaving radiance in SWIR bands to separate atmospheric signal, and introduce adjacency effect correction to address land–water boundary contamination. As a result, they often achieve a better performance in complex atmospheric conditions compared to other general approaches.

Therefore, we can conclude that UAV-RRS, as a quasi-ground *R_rs_* measurement method, offers greater flexibility compared to ground-based in situ measurements. It is capable of acquiring rich datasets in a short period, making it possible to conduct large-scale satellite spectral validation and comparison.

### 4.3. Strengths of UAV-RRS in Water Spectral Monitoring

The development and validation of UAV-RRS represents a significant advancement in water quality monitoring capabilities, comparing to traditional in situ measurements.

Unlike point-based in situ measurements, UAV-RRS provides continuous spatial coverage, revealing patterns and transitions that might be missed by discrete sampling. In addition, with meter-level resolution, UAV-RRS can detect small-scale features and heterogeneity in water optical properties that satellite sensors would average out. The system can be deployed rapidly and repeatedly, enabling monitoring at optimal times (e.g., during algal blooms or pollution events) or under specific conditions (e.g., during satellite overpass for validation). While not as inexpensive as satellite data, UAV-RRS offers a more affordable alternative to extensive boat-based sampling campaigns, especially for the routine monitoring of small water bodies. These advantages make UAV-RRS particularly valuable for monitoring small to medium-sized water bodies, which are often critical for local water supplies and ecosystem services.

## 5. Conclusions

To overcome the challenge of correcting rich in situ *R_rs_* data for water color remote sensing, as well as monitoring the water spectra of fine-scale water bodies, this study developed a hyperspectral *R_rs_* measurement system based on a UAV platform, called UAV-RRS.

The system underwent rigorous radiometric calibration outdoors using a standard diffuse reflectance plate. The UAV-RRS system achieved excellent radiometric stability with calibration errors consistently below 4% across the visible spectrum, demonstrating its reliability for quantitative water color measurements. To verify the system’s actual measurement performance, a ground–air-space-synchronized validation and application experiment was conducted in the middle reaches of Qiantang River, where spectral accuracy was tested. UAV-derived *R_rs_* measurements showed strong agreement with traditional ASD FieldSpec measurements (r > 0.9 in visible bands). The experiment showed that the near-ground UAV flight spectral data could achieve an accuracy comparable to ground-based measurements. Continuous spectral mapping along the middle reach of Qiantang River revealed significant spatial heterogeneity in water optical properties, with distinct upstream–downstream patterns that would be difficult and inconvenient to characterize using traditional sampling methods.

Additionally, the comparison between UAV–satellite *R_rs_* data indicated that the UAV-RRS system, under specific conditions, outperformed satellite remote sensing in both spatial and spectral accuracy. Comparison with Sentinel-2 data processed using seven atmospheric correction algorithms revealed notable differences in spatial detail and spectral accuracy. UAV-RRS captured finer spatial gradients in water optical properties compared to satellite measurements, highlighting the limitations of satellite remote sensing for small water bodies. These conclusions emphasize the complementary nature of UAV-based systems within the broader water color remote-sensing toolkit. While satellite observations provide unmatched coverage and temporal consistency, UAV platforms excel in capturing fine-scale spatial patterns and can be strategically deployed to study specific features or events.

The UAV-RRS system provides a new approach for the fine-scale water color remote sensing of water bodies, offering a practical solution for high-resolution spectral mapping of inland waters. Its ability to capture spatial heterogeneity at scales relevant to local water management makes it suitable for monitoring small water bodies.

Future work should focus on further miniaturizing the system components to reduce payload weight, extending flight duration, and developing automated data processing workflows to streamline the transition from raw measurements to actionable water quality information. Integration of UAV-RRS with in situ water sampling and satellite observations within a unified monitoring framework also represents a promising direction for comprehensive water quality assessment across multiple spatial and temporal scales.

## Figures and Tables

**Table 1:** Sensor optical and technical specifications of the Spectral Nexus GCS300 Spectrometers.

Parameter	Value/Description
Flight altitude	5–20 m above water surface
Flight speed	5 m/s approximately
Flight pattern	Manual operation with predefined routes aligned with river geometries
Sampling time	0.5–1 s per measurement point
Spatial resolution	<10 m between measurement points
Data acquisition window	9:00–15:00 local time (stable lighting conditions)
Flight duration	20–30 min per battery
Coverage per flight	6–9 km of watershed length
Integration time	1–20 ms (configurable based on illumination)
Data transmission	Real-time to ground station via 2.4 GHz link
Positioning accuracy	Horizontal: ±2 cm, Vertical: ±3 cm with RTK enabled

**Table 2:** Atmospheric correction algorithms for retrieving water surface reflectance.

AC Algorithm	Access Method
C2RCC	C2RCC is embedded in SNAP 10.0.0 software.
FLAASH	FLAASH is a tool in ENVI 5.6; the water retrieval option should be applied using band 9 (945 nm).
6S	6S is open-source; a Python 3.7 interface called Py6S can be used, see https://py6s.readthedocs.io (accessed on 29 April 2025), and it is wrapped into a Python batch process program, see https://github.com/Zhaoguanhua/AtmosphericCorrection (accessed on 29 April 2025).
Acolite	Acolite is an open-source Python program, with a binary package distributed also, see https://github.com/acolite/acolite (accessed on 29 April 2025).
SWIR-AC	Detailed algorithm is provided in Wang et al. [48].
iCor	iCor is a tool can be embedded into SNAP, see https://remotesensing.vito.be/services/icor (accessed on 29 April 2025). The AOT estimation algorithm in iCor cannot be applied in the water area, so it should be muted and manually specified (here, we use 0.1).
Sen2Cor	Sen2Cor is embedded in SNAP software and it is officially used to produce the L2A level product by the ESA.

**Figure A1:**
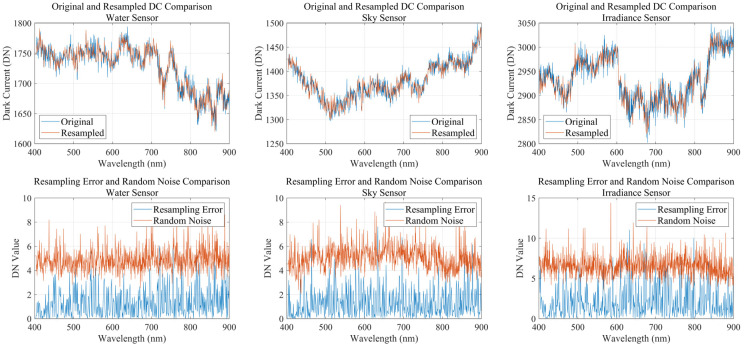
Original and resampled dark current comparison.

## Data Availability

The data will be made available upon request. Researchers interested in accessing the data may contact the authors.

## Supplementary Materials

The following supporting information can be downloaded at https://www.mdpi.com/article/10.3390/s25092879/s1, Table S1: GCS300 Series Spectrometer Technical Specifications.

## Abbreviations

The following abbreviations are used in this manuscript:

*R_rs_*	Remote-sensing reflectance
IOPs	Inherent optical properties
AOPs	Apparent optical properties
*a*	Absorption coefficient
*b_b_*	Backscattering coefficient
ASD	Analytical Spectral Devices
SBA	Skylight-blocked approach
UAV	Unmanned aerial vehicle
DN	Digital number
UAV-RRS	UAV-based *R_rs_* measurement system
IMU	Inertial measurement unit
SNR	Signal-to-noise ratio
CCD	Charge-coupled device
SD	Standard deviation
MSE	Mean squared error
CV	Coefficient of variation
ESA	European Space Agency
MSI	Multispectral instrument
NIR	Near-infrared
SWIR	Shortwave infrared
TOA	Top-of-atmosphere
BOA	Bottom of atmosphere
SRF	Spectral response function
PCC	Pearson correlation coefficient
RMSE	Root mean squared error
MAD	Mean absolute deviation
MRE	Mean relative error
SSIM	Structural similarity index

## Appendix A

### Appendix A.1. Resampled Dark Current Error Evaluation

In this study, we applied cubic spline interpolation to resample spectral data at 1 nm intervals. In Appendix A, we compare the interpolation-induced resampling error with typical random signal noise observed in practical measurements, as shown in Figure A1.

To verify the resampling error, we selected dark current measurements taken by three spectrometers at an integration time of 1 ms. These measurements were then resampled using cubic spline interpolation. Since the true values for the resampled wavelengths cannot be measured directly, we used linear interpolation of the original spectrum as a substitute. The error between the cubic spline interpolation and linear interpolation at each wavelength serves as an approximation of the resampling error. The standard deviation between multiple measurements of the dark current can be considered as random noise. The comparison between the two is shown in the second row of Figure A1. From the analysis, it can be observed that the resampling error is smaller than the random noise, indicating that the effect of resampling on the results is negligible.
