# Supplementary material for: Design and Construction of UAV-Based Measurement System for Water Hyperspectral Remote-Sensing Reflectance
Source: Sensors (Basel). 2025 May 2;25(9):2879. doi: 10.3390/s25092879 (PMC12074439; doi:10.3390/s25092879)
Supplement: Supplementary file 1 [file sensors-25-02879-s001.zip › sensors-3556503-supplementary.pdf]

Supplementary S1

Supplementary S1.1 Introduction of the GCS300 series spectrometer

The GCS300 series spectrometer is a newly developed general-purpose spectrometer based on holographic flat-field concave diffraction grating technology. Compared to traditional planar grating spectrometers, the GCS300 offers several advantages, including low stray light, reduced readout noise, high diffraction efficiency, and minimal temperature sensitivity. It employs a high-performance linear array detector from HAMAMATSU, ensuring an improved signal-to-noise ratio and wide dynamic range.

In addition, the spectrometer supports spectral analysis software and provides a secondary development kit, enabling users to quickly build and customize their own spectral application systems.

Key features of the GCS300 series include:

1. Holographic flat-field concave grating for effective stray light reduction.
2. Equipped with a high-performance linear array detector from HAMAMATSU.
3. Built-in high-order optical filters to suppress second- and third-order spectral interference.
4. Multiple communication interfaces, including USB 2.0 (480 Mbps) and RS232.
5. Support for various trigger signal modes, allowing control of external light sources and synchronization.
6. Multiple slit width options with easy replacement design.

The detailed technical specifications are listed in Table S1.

Table S1. GCS300 Series Spectrometer Technical Specifications

**Technical Specifications**	**Value**
Product model	GCS 300 VIS-NIR
Detector type	HAMAMATSU Photodiode Array
Pixel	2048
Grating	Holographic flat-field concave diffraction grating
Slit	Optional slit widths (25 μm, 50 μm, 100 μm, 200 μm)
Spectral range	VIS-NIR: 350–950 nm
Pixel resolution	0.4 nm
Optical resolution	1.5 nm (50 μm slit)
Wavelength accuracy	±0.2 nm
Stray light	<0.05% @400 nm
SNR	10000:1
AD resolution	16 bit
Integration time	10 μs–60 s
Power supply	5V/220mA
Size	127 mm×106 mm×57 mm
Communication interface	USB 2.0 (480 Mbps), RS232
Expansion interfaces	SICS-26 (I2C, SPI, GPIO, RS232)
Spectrum software	*SpectraNexus* Spectral analysis software
Secondary development	*SpectraArsenal* software development kit (SDK)
